# Rapid measurement of 8-oxo-7,8-dihydro-2′-deoxyguanosine in human biological matrices using ultra-high-performance liquid chromatography–tandem mass spectrometry

**DOI:** 10.1016/j.freeradbiomed.2012.03.004

**Published:** 2012-05-15

**Authors:** Patricia M.W. Lam, Vilas Mistry, Timothy H. Marczylo, Justin C. Konje, Mark D. Evans, Marcus S. Cooke

**Affiliations:** a Oxidative Stress Group, Department of Cancer Studies and Molecular Medicine, University of Leicester, Leicester LE2 7LX, UK; b Endocannabinoid Research Group, Reproductive Sciences Section, Department of Cancer Studies and Molecular Medicine, University of Leicester, Leicester LE2 7LX, UK; c Department of Genetics, University of Leicester, Leicester LE2 7LX, UK

**Keywords:** ROS, reactive oxygen species, 8-oxodG, 8-oxo-7,8-dihydro-2′-deoxyguanosine, HPLC, high-performance liquid chromatography, RSD, relative standard deviation, SPE, solid-phase extraction, UHPLC, ultra-high-performance liquid chromatography, LOD, limit of detection, LOQ, limit of quantification, 8-Oxo-7,8-dihydro-2′-deoxyguanosine, Mass spectrometry, Human, Biomarkers, Oxidative stress, DNA repair, Oxidatively damaged DNA, Urine, Free radicals

## Abstract

Interaction of reactive oxygen species with DNA results in a variety of modifications, including 8-oxo-7,8-dihydro-2′-deoxyguanosine (8-oxodG), which has been extensively studied as a biomarker of oxidative stress. Oxidative stress is implicated in a number of pathophysiological processes relevant to obstetrics and gynecology; however, there is a lack of understanding as to the precise role of oxidative stress in these processes. We aimed to develop a rapid, validated assay for the accurate quantification of 8-oxodG in human urine using solid-phase extraction and ultra-high-performance liquid chromatography–tandem mass spectrometry (UHPLC–MS/MS) and then investigate the levels of 8-oxodG in several fluids of interest to obstetrics and gynecology. Using UHPLC–MS/MS, 8-oxodG eluted after 3.94 min with an RSD for 15 injections of 0.07%. The method was linear between 0.95 and 95 nmol/L with LOD and LOQ of 5 and 25 fmol on-column, respectively. Accuracy and precision were 98.7–101.0 and <10%, respectively, over three concentrations of 8-oxodG. Recovery from urine was 88% with intra- and interday variations of 4.0 and 10.2%, respectively. LOQ from urine was 0.9 pmol/ml. Rank order from the greatest to lowest 8-oxodG concentration was urine>seminal plasma>amniotic fluid>plasma>serum>peritoneal fluid, and it was not detected in saliva. Urine concentrations normalized to creatinine (*n*=15) ranged between 0.55 and 1.95 pmol/μmol creatinine. We describe, for the first time, 8-oxodG concentrations in human seminal plasma, peritoneal fluid, amniotic fluid, and breast milk, as well as in urine, plasma, and serum, using a rapid UHPLC–MS/MS method that will further facilitate biomonitoring of oxidative stress.

## Highlights

► We report a novel, rapid solid-phase extraction UHPLC–MS/MS method for urinary 8-oxodG. ► We examined 8-oxodG in biological matrices of interest to reproductive medicine. ► We quantified 8-oxodG in plasma, serum, and seminal, amniotic, and peritoneal fluids. ► Oxidative stress can be accurately and rapidly quantified in clinical samples.

## Introduction

Oxidative stress is the imbalance between the production and the elimination of reactive oxygen species (ROS), in favor of the former [1]. Increasing evidence has implicated oxidative stress in the pathogenesis of numerous major diseases including Parkinson, Alzheimer, atherosclerosis, and cancer (reviewed in [2]). Interaction of ROS with DNA can lead to the modification of the constituent 2′-deoxyribonucleosides to produce a range of oxidation products, for example, 8-oxo-7,8-dihydro-2′-deoxyguanosine (8-oxodG [2]). In addition to being mutagenic, 8-oxodG may have other detrimental effects on cell function, including promotion of microsatellite instability and acceleration of telomere shortening [3]. Highlighting the biological significance of 8-oxodG is a system of multiple, highly redundant repair pathways that prevent the persistence of damage in DNA and the 2′-deoxyribonucleotide pool [4]. In humans, the products of repair are thought to generate 8-oxo-7,8-dihydroguanine (8-oxoGua), predominantly from DNA, and 8-oxodG from the 2′-deoxyribonucleotide pool, which are excreted finally into urine. Early reports of a dietary contribution to urinary levels of nucleobase products, such as 8-oxoGua [5,6], provided the basis for the overwhelming focus on examining modified 2′-deoxyribonucleosides, and 8-oxodG in particular. Whereas genomic DNA measurements of oxidative stress biomarkers have been shown to be at risk of artifactual formation during sample extraction and workup [7], these risks are minimal for urinary measurements of similar markers [8]. Nevertheless, there are profound differences in the levels of urinary 8-oxodG determined by chromatographic techniques, compared to immunoassay [9].

Urine is an increasingly popular biological matrix in which 8-oxodG is measured, because of the noninvasive collection and lessened ethical issues, and allows access to vulnerable groups and archived samples. The analytical methods applied to measuring urinary 8-oxodG can be divided into two main types: chromatographic and immunoassay. The former includes HPLC in conjunction with tandem mass spectrometric (HPLC–MS/MS [10]) or electrochemical detection [11]. However, increasingly the strengths of mass spectrometry are being exploited; these include high sensitivity, specificity, and the ability to measure numerous lesions in a single run. Additionally, the use of a stable isotopically labeled internal standard can account for loss during sample preparation and ion suppression and be used for absolute quantification. The main disadvantages of mass spectrometric techniques are the high cost of equipment and the need for skilled staff for operation and maintenance. As urine is a complex matrix, a cleanup step is often performed to facilitate detection and maximize equipment lifetime. Solid-phase extraction (SPE) has been employed as a relatively fast and simple way to extract and clean up 8-oxodG from urine before mass spectrometric detection [12,13]. Our laboratory has increasingly moved toward HPLC–MS/MS, combined with SPE, for urinary 8-oxodG analysis [13,14]. Here, we report the development of a faster method of detecting 8-oxodG in urine, and other biomatrices, which exploits ultra-high-performance liquid chromatography–electrospray ionization–tandem mass spectrometry (UHPLC–ESI–MS/MS).

There is an increasing awareness of the role of oxidative stress in pregnancy and fertility [15]. It is widely recognized that pregnancy is a state of “physiological” oxidative stress [16] secondary to increased metabolic activity in the placental mitochondria [17]. Additionally, oxidative stress has been implicated in female infertility, preeclampsia [18,19], gestational diabetes [20], preterm labor, polycystic ovarian disease, endometriosis [21], and fetal growth restriction (FGR) [22,23]. However, there is a lack of basic research in understanding how oxidative stress can have an impact on reproduction. Our first step toward this goal is the development and validation of a robust UHPLC–ESI–MS/MS assay for 8-oxodG in urine, plasma, serum, and reproductive fluids.

## Materials and methods

### Chemicals

HPLC-grade methanol and water were purchased from Fisher (Loughborough, UK) and water of HPLC grade was obtained from an water purification system (Maxima ELGA). 8-OxodG (powder form; purity ≥98%) and formic acid (mass spectrometry grade) were from Sigma–Aldrich (Poole, UK). 8-OxodG was reconstituted in sterile ultrapure water to a stock concentration of 5 pmol/μl. Aliquots were stored at −80 °C until required. Isotopically labeled internal standard (IS; 8-[^15^N_5_]oxodG) was synthesized, as reported by Singh et al. [24], and aliquots of 183 μM 8-[^15^N_5_]oxodG were stored at −80 °C until use. When required, these were diluted to 2 pmol/μl in HPLC-grade water.

### Sample collection

Healthy volunteers (seven females, eight males; ages 22–61) were recruited at random from staff and students of the University of Leicester to provide urine samples. Approval had been granted by the University of Leicester, College of Medicine, Biological Sciences and Psychology Non-Clinical Ethics Committee. Ethical approval for the collection of samples from patients had been granted by the Leicestershire, Northamptonshire, and Rutland Research Ethics Committee 1.

All volunteers gave informed, written consent to take part in the study. The biological matrices investigated were adult plasma, serum, urine, saliva, breast milk, peritoneal fluid, amniotic fluid, and seminal fluid. All collected fluids were stored at −80 °C until required. Whole blood (approximately 4 ml) was collected into EDTA tubes (Sarstedt Ltd., Leicester, UK) or serum gel monovettes (Sarstedt). Blood was transported and maintained on ice, until centrifugation within 30 min of collection at 1200*g* for 30 min at 4 °C to separate either plasma or serum from cells. Aliquots of plasma or serum (0.5 ml) were then transferred to clean 7-ml Kimble vials (Kinesis, St. Neots, Cambridgeshire, UK). Amniotic fluid was collected at elective cesarean section into sterile universal tubes and transported to the laboratory where it was immediately transferred to 7-ml Kimble vials and kept on ice before storage. Breast milk was collected from breast-feeding mothers using a breast pump, 1–4 weeks after birth of their child. Breast milk was centrifuged at 1200*g* for 30 min at 4 °C and the supernatant transferred to Kimble scintillation vials. Midstream urine samples were collected from 21 volunteers and aliquotted into Kimble vials before storage at −80 °C. Six samples were used for assay validation and the remaining used to investigate the consistency of 8-oxodG concentration with previous publications. Creatinine levels were determined using a commercially available assay (DetectX human urinary creatinine kit; Arbor Assays, Ann Arbor, MI, USA). Saliva was collected from six healthy smokers, with no signs of gum disease (four female, two male) using Salivettes (Sarstedt). Saliva was isolated by centrifugation (1200*g* for 30 min at 4 °C) and transferred to 7-ml vials. Peritoneal fluid was collected from seven women during laparoscopy, transported on ice, and centrifuged at 1200*g* for 30 min at 4 °C, and the supernatant was transferred to 7-ml Kimble vials.

Seminal fluid was obtained from men attending the Andrology Unit (Leicester Royal Infirmary) for routine semen analysis. Seminal fluid was collected by masturbation, and samples were allowed to liquefy at room temperature for 1 h and then transported to the laboratory on ice, within 2 h of production. Seminal fluid was transferred into 7-ml Kimble vials and centrifuged at 1200*g* for 30 min at 4 °C to separate seminal plasma from spermatozoa and cellular debris. Supernatants were transferred into clean vials.

### Solid-phase extraction of 8-oxodG from human biomatrices

Samples were thawed on the day of extraction and then plasma, serum, peritoneal fluid, urine, and amniotic fluids were spun at 16,000*g* for 10 min and saliva was spun at 3000*g* for 10 min at 4 °C. Plasma (1 ml), serum (1 ml), urine (0.5 ml), saliva (1 ml), amniotic fluid (1 ml), breast milk (1 ml), peritoneal fluid (1 ml), and seminal plasma (0.5 ml) were then spiked with 10 pmol of 8-[^15^N_5_]oxodG internal standard. Plasma, serum, urine, amniotic fluid, and peritoneal fluid were diluted 1:1 with deionized water, whereas breast milk and saliva were diluted 1:1 with 5% (w/v) phosphoric acid. Seminal plasma was diluted with 5% (w/v) phosphoric acid up to a total volume of 2 ml. Samples were then microcentrifuged at 16,000*g* for 1 min at room temperature. Env+Isolute (1 ml, 50 mg) cartridges (Biotage, Uppsala, Sweden) were preconditioned with 1 ml methanol and 1 ml H_2_O. Samples were introduced onto the cartridges and drawn through at a flow rate of approximately 1 ml/min. The cartridges were washed twice with 300 μl H_2_O and 8-oxodG was eluted in 2 μl×300 μl of 20% (v/v) acetonitrile in methanol. The eluents were dried under nitrogen before reconstitution in 50 μl mobile phase and centrifuged at 1000*g* for 1 min before transferring to Eppendorf tubes. Samples were then centrifuged at 16,000*g* for 10 min before the supernatants were transferred to HPLC vials.

To determine the extraction efficiency, urine collected from one healthy individual was centrifuged at 16,100*g* at 4 °C for 10 min. The recovery of 8-oxodG was assessed by the addition of five different amounts of 8-oxodG (0, 5, 10, 15, and 20 pmol) to 500 μl of the urine supernatant, before SPE. In parallel, 5 500-μl aliquots of the same urine supernatant underwent SPE; however, these were spiked with the same amount of 8-oxodG after SPE. All 10 samples were spiked with 10 pmol IS after SPE. The peak area ratio (PAR) of unlabeled 8-oxodG to IS in all 10 samples was calculated. The percentage recovery of 8-oxodG was then calculated as (PAR_Pre_/PAR_Post_)×100%, where PAR_Pre_ represents samples with the addition of unlabeled 8-oxodG before SPE, and PAR_Post_ represents samples with the addition of unlabeled 8oxodG after SPE.

### UHPLC–MS/MS analysis

The UHPLC–ESI–MS/MS system comprised an Acquity UPLC in line with a Quattro Premier tandem mass spectrometer (Waters, Elstree, UK). The column was an Acquity UPLC BEH C_18_ (2.1 mm×100 mm) maintained at 40 °C. An isocratic mobile phase of 5% methanol, 0.1% formic acid was used with a flow rate of 0.25 ml/min. Samples were maintained at 4 °C throughout. Analytes were quantified using tandem electrospray ionization mass spectrometry in positive-ion mode. Source parameters were capillary voltage 3.2 kV, cone voltage 20 V, source temperature 110 °C, desolvation temperature 350 °C, cone gas flow 50 L/h, desolvation gas flow 800 L/h. MS/MS conditions for each precursor [M+H]^+^ ion comprised entry, collision, and exit energies of 2, 15, and 0 eV, respectively. Product ions were monitored in multiple-reaction monitoring mode. Mass transitions were 8-oxodG (*m*/*z* 284>167.9) and 8-[^15^N_5_]oxodG (*m*/*z* 289>173). A secondary or qualifier transition (*m*/*z* 284>116.8) was used to confirm the identity of 8-oxodG in samples [25]. Injection volume was 5 μl. Seven-point calibration curves spiked with IS were generated. Spectrograms were integrated using MassLynx software version 4.1. QuanLynx software calculated the concentration of 8-oxodG using calibration curves of concentration against relative response calculated as follows:

relativeresponse(y)=(peakarea)/(ISarea/[IS]).

An external calibration curve, in which values were calculated using the internal standard, was used initially because our internal standard had not been synthesized under GMP conditions, potentially affecting accurate quantification. However, once the value of the internal standard had been verified using external reference standards, we could use a standard isotope dilution method, with a known amount of internal standard added to each sample, and the samples were quantified with reference to the internal standard.

### Validation of UHPLC–ESI–MS/MS method for 8-oxodG

The UHPLC–MS/MS method was validated according to FDA guidelines (http://www.fda.gov). Linearity of the assay was determined using 15 seven-point standard curves (4.75–475 fmol on-column for 8-oxodG) and linear regression analysis. Consistency of retention time was investigated after 15 injections of 95 fmol 8-oxodG. Accuracy was calculated for three concentrations of 8-oxodG (9.5, 95, and 237.5 fmol on-column). Precision was calculated after 15 repeat injections of 1.9–95 nmol/L 8-oxodG standards in water (9.5–475 fmol 8-oxodG on-column). Limits of quantification and detection were defined as 8-[^15^N_5_]oxodG responses, which yielded signal-to-noise ratios, without smoothing, of greater than 10 and greater than 3, respectively, and were calculated both for nonextracted standards of 8-[^15^N_5_]oxodG in mobile phase and after extraction from human urine spiked with 8-[^15^N_5_]oxodG. We used isotopically labeled IS for these studies to overcome the complication of endogenous 8-oxodG in the urine samples. Intra- (*n*=3) and interday (*n*=9) precision and accuracy data of urinary 8-oxodG over 3 days were calculated from pooled urine (0.5-ml aliquots) This was determined by spiking 0.5-ml aliquots of urine with 8-oxodG (5, 20, and 50 pmol) and IS (10 pmol). Accuracy was presented as the percentage of measured 8-oxodG concentrations to expected values. Ion suppression was determined for measurement of IS in urine. To achieve this, the response observed for IS spiked into sample after SPE was compared with the response observed for a nonextracted standard of the same concentration.

We employed an external calibration curve for quantification of 8-oxodG in these studies, to investigate whether the presence of matrix has any effect on the quantification of samples. Identical standard curves were generated in diluted urine (0.125 ml made up to 1 ml with water) and plasma (1 ml) to mimic the extraction conditions. Furthermore, to demonstrate that shifting of these calibration curves is a consequence of the background 8-oxodG concentration in these samples, the same samples were extracted using the normal protocol (*n*=4) described above and the observed concentrations were compared to the derived concentrations from the differences in *y*-axis intercepts of the calibration curves generated in the presence or absence of biofluid.

To further verify the presence of 8-oxodG each sample was also analyzed using a second transition for 8-oxodG (*m*/*z* 284>116.8). The established ratio ((*m*/*z* 284>116.8):(*m*/*z* 284>167.9)) of the peak areas observed for the two transitions employed after analysis of standards (4.75–475 fmol on-column for 8-oxodG) over 7 days (*n*=49) could then be used to set the acceptable permitted range for the 8-oxodG relative ion intensity in samples, according to European Commission Council Directive 96/23/EC;SANCO/1805/2000^1^. Samples not conforming to these permitted tolerances were omitted from the data sets.

### Statistical analysis

The validation data are presented as the mean±SD. Relative standard deviation was calculated as (standard deviation/mean)×100%. Statistical comparison of 8-oxodG levels in different biological matrices was achieved using a paired student *t* test.

## Results

### Validation

**Fig. 1:**
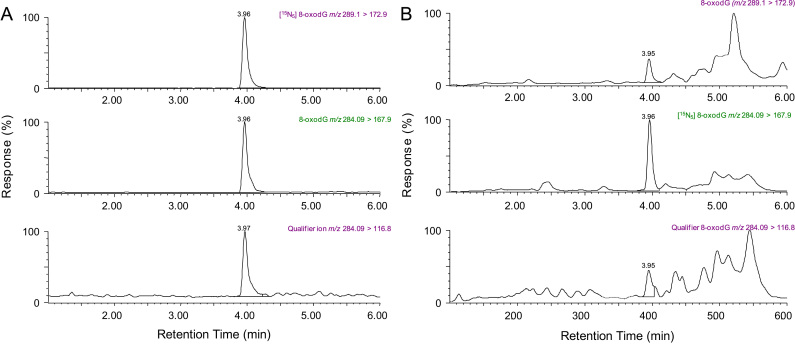
(A) Representative spectrograms of 8-oxodG standard (*m*/*z* 284.09>167.9), corresponding qualifier ion (*m*/*z* 284.09>116.8), and internal standard 8-[^15^N_5_]oxodG (*m*/*z* 289.1>172.9). (B) Representative chromatogram from human urine sample displaying detectable levels of 8-oxodG and internal standard.

8-OxodG had a retention time of 3.96±0.003 min (Fig. 1A) and a relative standard deviation (RSD) of 0.07%. As an endogenous component of urine, we could not perform extractions in the absence of 8-oxodG in authentic matrix (Fig. 1B); however, no peaks were observed for the IS in nonspiked samples. The qualifier ion (*m*/*z* 284>116.8) was used in the analysis of 8-oxodG to confirm the identity of 8-oxodG peaks particularly when concentrations were low.

**Fig. 2:**
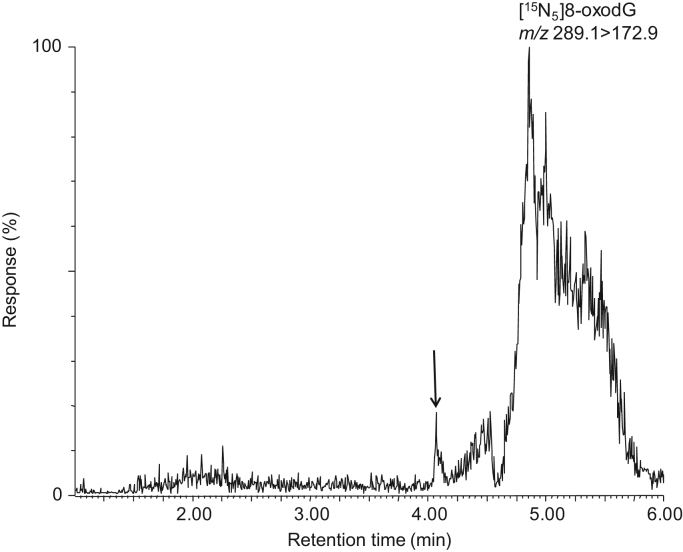
Mass spectrograms showing LOQ of 8-oxodG in extracted urine. The internal standard, 8-[^15^N_5_]oxodG (*m*/*z* 289.1>172.9) was used as a surrogate for 8-oxodG to determine the LOQ for urine because of the endogenous levels of 8-oxodG present in all urine samples. For LOQ, 0.5 ml of urine was spiked with 450 fmol of 8-[^15^N_5_]oxodG and extracted using SPE as described under materials and methods. The spectrogram is representative of triplicates injected on three separate occasions and has not been smoothed.

Linearity was derived from 15 calibration curves (Table 1) and responses were linear over the nonextracted 8-oxodG concentration range of 4.75–475 fmol on-column (equivalent to 0.95–95 nmol/L). Precision data measured in repeat injections of 1.9–95 nmol/L 8-oxodG standards on-column are shown in Table 1 and have RSD values of less than 10%. The accuracy (Table 1) for differing amounts of 8-oxodG ranged from 98.7% to 101.0% (derived from 9.5, 95, and 237.5 fmol on-column). The LOD for the nonextracted standard was 5 fmol, and LOQ was 25 fmol, on-column (Fig. 2). Average recovery from 0.5 ml urine was 88% with intra- and interday variabilities of 4.0 and 10.2%, respectively.

Intra- and inter-day precision data for 8-oxodG extracted from urine (Table 2) were deemed acceptable with intra- and interday RSD values ranging from 4.4% to 11.1% and 5.2% to 12.7%, respectively. Both intra- and inter-day accuracies were good, with less than 15% variability from expected measurements. LOD and LOQ were calculated using IS. IS extracted from 0.5 ml human urine yielded LOQ values of 0.9 pmol/ml (signal-to-noise ratio >10).

Ion suppression values were determined by postextraction addition of IS followed by comparison of peak area obtained with the peak area of nonextracted IS of the same concentration. Ion suppression varied both between biomatrices and between individuals. Biofluids in the order of least to most ion suppression after SPE were plasma (0%)<serum (<11%)<amniotic fluid (<40%)<saliva (49–55%)<peritoneal fluid (<65%)<breast milk (22–75%)<urine (30–90%)<seminal plasma (>90%). The high levels of ion suppression in urine prompted us to investigate various volumes for extraction (0.5, 0.25, and 0.125 ml; *n*=6). Ion suppression was proportional to the volume of urine employed for the extraction (78%, 38%, 0%, respectively) but 8-oxodG concentration was unaffected (14.44±0.50, 14.06±0.43, and 15.14±0.58 nM, respectively). Consequently, 0.125 ml urine was employed for all further analyses.

**Fig. 3:**
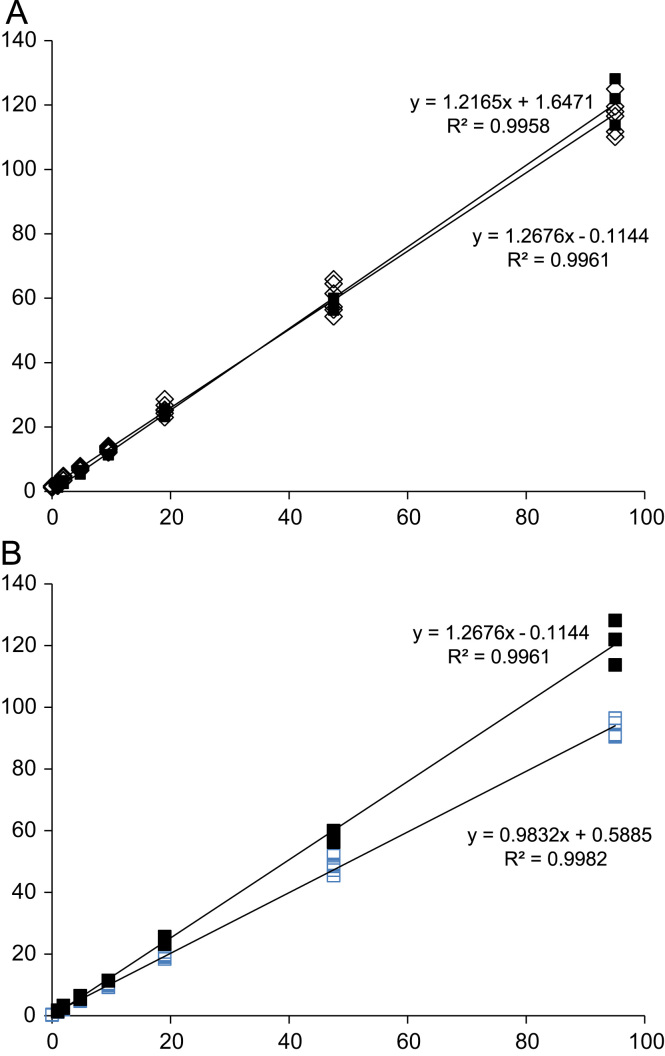
Comparison of calibration curves generated in water (■) and (A) human urine (◊) or (B) human plasma (□).

When calibration curves were generated in water, diluted urine, and plasma on the same day, the corresponding linear regression analyses yielded the equations *y*=1.2676*x*−0.1144, *y*=1.2165*x*+1.6471, and *y*=0.9832*x*+0.5885, respectively, and linear correlation coefficients (*R*^2^) of 0.996, 0.996, and 0.998 (Fig. 3). When the differences in *y*-intercept (response) are converted to concentrations (nM) using the equation of the water standard, taking into account the dilution of the urine, we obtain concentrations of 0.65 and 8.11 nM for plasma and urine, respectively. Urine and plasma analyzed on the same day (*n*=4) yielded concentrations of 0.68±0.21 and 6.93±0.88 nM, respectively.

The mean relative ion intensity for 8-oxodG after analysis of standards was 0.125. According to European Commission Council Directive 96/23/EC;SANCO/1805/2000 the allowable tolerance for the relative ion intensity is ±30%, giving an acceptable range of 0.087–0.162. When calibration curves were generated in urine and plasma samples as described above, the relative ion intensities (mean±SD) were 0.110±0.017 and 0.111±0.021, respectively.

### Levels of 8-oxodG in human biofluids

The validated SPE and UHPLC–ESI–MS/MS methods were employed subsequently to identify and quantify 8-oxodG in a range of human biofluids. Levels of 8-oxodG greater than the LOD were present in all biological matrices tested, with the exception of saliva (Table 3). All remaining samples passed our exclusion criteria in terms of coelution with 8-[^15^N_5_]oxodG and with relative ion intensities for the two 8-oxodG transitions within the agreed allowable tolerance. Relative ion intensities (mean±SD) were urine, 0.115±0.018; plasma, 0.121±0.018; serum, 0.132±0.028; breast milk, 0.125±0.019; amniotic fluid, 0.128±0.023; and peritoneal fluid, 0.113±0.023.

The highest mean level of 8-oxodG was found in urine, which also had the largest range, with the lowest levels of 8-oxodG in peritoneal fluid. Mean levels of 8-oxodG were similar in both plasma and serum. Rank order concentrations of 8-oxodG in biological matrices were urine>seminal plasma>amniotic fluid>plasma>serum>peritoneal fluid (Table 3). Normalization of urinary 8-oxodG levels to creatinine content (*n*=15) gave a range of concentrations of 0.55–1.95 pmol/μmol creatinine and a mean±SD of 1.10±0.39 pmol/μmol creatinine.

## Discussion

We have developed and validated a rapid UHPLC–ESI–MS/MS method with simple SPE to quantify 8-oxodG in a number of human biomatrices. To the best of our knowledge, this is the first report describing an assay to determine levels of 8-oxodG in a range of reproductive fluids. Indeed, there are only two reports that describe the use of UHPLC for 8-oxodG, in urine [25] and DNA [26]. The former is comparable to our method, in terms of LOQ, precision, accuracy, and range, but has a fourfold longer run time (23 min) and requires postcolumn incorporation of acetonitrile to aid ionization. Our UHPLC method was also faster than previous HPLC methods, which ranged from 10 min [27,28] to ≥1 h, including column reconditioning [10,29]. The sensitivity and robustness of our method compare favorably with previously published methods, e.g., LOD and LOQ values of 5 and 25 fmol on-column, respectively, versus 5 and 20 fmol, respectively [29].

The short run-time of our UHPLC method increases throughput and decreases solvent costs compared with “classical” HPLC and has the potential for full automation. Recovery of 8-oxodG after SPE was 88%, which is similar to that reported elsewhere using Waters Oasis HLB cartridges [29,30]. These levels of recovery are very good and do not represent a significant decrease compared with methods that simply dilute urine in buffer before injection (e.g., 107% [25], 96% [27], >97% [28]). Direct injection methods can be affected by ion suppression, the presence of undesirable additional peaks, and noisy baselines that may affect the specificity and sensitivity. We employed a qualifier transition and incorporated an acceptable tolerance limit for the acceptance of data to decrease the possibility of misidentification of the 8-oxodG and of the contribution of any underlying peak. In some urine samples, we observed peaks that were close to the retention time of authentic 8-oxodG and required the use of the qualifier to verify 8-oxodG identity. The relative ratios between qualifier and quantifier ions were within the acceptable tolerance (see above) and the use of relative ion ratios greatly improved the confidence in the results and aided in the detection of any underlying peaks, which would otherwise distort the results.

A number of biomatrices demonstrated significant ion suppression that was sample dependent, e.g., ion suppression in peritoneal fluid samples varied from nil to 65% and in urine from 30% to 90%. This did not overtly affect the accuracy and precision of our method and was decreased, without changes to measured 8-oxodG concentration, by extracting from smaller volumes. When different volumes of urine were extracted (*n*=3) no significant changes were observed in the mean relative ratio of quantifier to qualifier ion (0.5 ml, 0.088; 0.25 ml, 0.098; 0.125 ml, 0.103). It is possible that preassessment and dilution of samples before extraction, using specific gravity, could increase the robustness of this method further and increase the lifetime of the UHPLC column. Qualitative assessment of ion suppression has been previously investigated only by Henriksen et al. [25]. Using their UHPLC system, 0.11-ml urine samples were responsible for 17% ion suppression, whereas we observed no ion suppression with similar (0.125 ml) urine volumes. Consequently, we recommend that for urinary 8-oxodG analysis the optimal volume is 0.125 ml or less, for which SPE can be used to remove components contributing to ion suppression.

To address possible questions over the accuracy of employing external calibration curves for quantification of 8-oxodG in biofluids, we created calibration curves in the presence and absence of urine and plasma and found that linearity was maintained (*R*^2^ values >0.99) and very little difference was observed between calibration curves produced in urine and water. The slope of the calibration curve in plasma was, however, slightly reduced compared with that in water. It is expected that differences in the *y*-intercepts of these curves will represent the concentration of 8-oxodG determined in these samples. Calculated values for urine (8.11 nM) are slightly higher (117%) than that predicted after analysis of the same sample by the methods detailed above (6.93 nM), whereas for plasma, the calculated value (0.65 nM) is an even closer match (95%) to the observed concentration (0.68 nM). Consequently, the use of an external standard curve is acceptable for the quantification of 8-oxodG.

Normalization of urinary 8-oxodG levels (*n*=15) gave a range of concentrations, 0.55–1.95 pmol/μmol creatinine, which is in good general agreement with the previously published normalized urinary 8-oxodG levels, e.g., 0.5–4.0 [29], 0.6–3.3 [28], and 0.7–4.2 pmol/μmol creatinine [31].

Free radicals are associated with normal reproductive physiology including ovulation, implantation, pregnancy maintenance, and parturition (reviewed in [21]). However, the origin of oxidative stress in complications of pregnancy is unknown and represents an increase over and above that of the so-called physiological oxidative stress of pregnancy [23]. A recent report described the retrospective measurement of urinary 8-oxodG, at two points in early pregnancy (∼12 and ∼28 weeks gestation), in women whose pregnancies were affected or unaffected by FGR [23]. A significant increase in urinary 8-oxodG was noted at both time points in the women whose pregnancies were diagnosed with FGR [23]. Here we demonstrate that 8-oxodG is present in amniotic fluid at term pregnancy. Amniotic fluid mainly comprises fluid excreted by the fetus, and as such levels of 8-oxodG may be indicative of fetal exposure to DNA-damaging agents. This warrants further investigation.

Oxidative stress is essential for sperm motility and both capacitation and the acrosome reaction, which are physiological changes necessary for fertilization [32–34]. This requirement for oxidative stress may explain the relatively high levels of 8-oxodG present in seminal plasma. Ours are the first data demonstrating the presence of 8-oxodG in seminal plasma, although 8-oxodG in sperm nuclear DNA is well documented, for example [35–37]. Despite the limited numbers of samples, the study of 8-oxodG levels in seminal fluid equally warrants further investigation. Not only is measurement of 8-oxodG simpler in seminal fluid than in DNA, there is less risk of artifactual generation of damage. Measurement of levels in seminal plasma would be particularly interesting in the context of male infertility, as increased levels of ROS have been demonstrated in semen samples of infertile men [38,39].

Previously, 8-oxodG has been quantified in human saliva in the 10–50 nM range using ELISA [30,40]. Using our UHPLC–ESI–MS/MS method we were unable to detect any 8-oxodG in saliva despite the predicted levels being markedly greater than the LOQ (1 nM) for this assay. It is possible that ion suppression by the complex saliva matrix is responsible for this; however, we found that there was no greater ion suppression in saliva than in the other matrices. This adds to previous concerns regarding the specificity of ELISA kits used for measurement of 8-oxodG and hence the levels of 8-oxodG reported in saliva [30].

We have developed a rapid SPE and UHPLC–ESI–MS/MS method to quantify 8-oxodG in urine as well as in a range of reproductive biological matrices. This assay will facilitate future studies investigating the role of oxidative stress in reproductive physiology and pathophysiology and the biomonitoring of oxidative stress in the fetus.

## Figures and Tables

**Table 1:** Validation parameters for the quantification of 8-oxodG by UHPLC–ESI–MS/MS.

Validation parameter	Result
Range	4.75–475 fmol
Linearity (*n*=15)	*y*=1.59±0.07 to 0.08±0.14; *R*^2^=0.998
Precision (*n*=6)	9.5 fmol: CV=7%
	95 fmol: CV=4%
	237.5 fmol: CV=4%
Accuracy (*n*=6)	9.5 fmol: 101.0±9.6%
	95 fmol: 100.3±4.5%
	237.5 fmol: 98.7±1.9%
LOD	8-[^15^N_5_]oxodG: 5 fmol on-column
LOQ	8-[^15^N_5_]oxodG: 25 fmol on-column
Urine SPE intraday (CV%)	4
Urine SPE interday (CV%)	10

**Table 2:** Accuracy of 8-oxodG quantification in urine after SPE.

	Amount of 8-oxodG added to urine (nM)			
	0	10	40	100
Intraday (*n*=3)				
Mean±SD (nM)	12.21±1.35	19.70±1.39	44.58±3.83	104.21±4.54
RSD (%)	11.1	7.0	8.6	4.4
Accuracy (%)	–	88.7	85.4	92.9

Interday (*n*=9)				
Mean±SD (nM)	11.15±1.42	18.20±1.64	45.41±3.09	101.93±5.34
RSD (%)	12.7	9.0	6.8	5.2
Accuracy (%)	–	86.0	88.8	91.7

**Table 3:** 8-OxodG concentrations in human biomatrices.

		8-oxodG (nM)	
Biological matrix	*n*	Mean±SD	Range
Urine	6	16.11±8.84	8.4–32.88
Saliva	4	ND	ND
Peritoneal fluid	5	0.07±0.03	0.04–0.11
Plasma	5	0.14±0.05	0.07–0.19
Serum	5	0.08±0.02	0.05–0.11
Amniotic fluid	5	0.46±0.22	0.27–0.85
Seminal plasma	4	3.00±0.96	1.57–3.70
Breast milk	3	0.92±0.25	0.63–1.20

ND, none detected.

## Footnotes

1 European Commission decision laying down performance criteria for the analytical methods to be used for detecting certain substances and residues thereof in live animals and animal products according to Council Directive 96/23/EC; SANCO/1805/2000.
